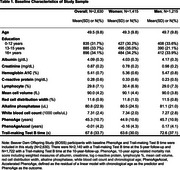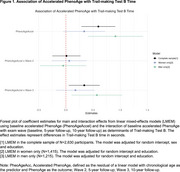# Accelerated Phenotypical Aging in Midlife is Associated with Long‐Term Cognitive Decline in Middle‐Aged Adults

**DOI:** 10.1002/alz70856_106183

**Published:** 2026-01-07

**Authors:** Natascha Merten, Mary Ryan Baumann, Adam J Paulsen, Huan Li, Richard J. Chappell, Yanjun Chen, Lindsay R Clark, Sterling C Johnson, James S Pankow, Art Walaszek, Madeleine G Soss

**Affiliations:** ^1^ University of Wisconsin‐Madison, Madison, WI, USA; ^2^ University of Minnesota, Minneapolis, MN, USA

## Abstract

**Background:**

PhenoAge is a multi‐system blood‐based aging marker that uses common clinical tests of glucose metabolism, inflammation and kidney and liver function. This easily‐obtained marker determines whether a person is younger or older on a biological and physiological level than expected by their chronological age. A higher PhenoAge is associated with increased risk of disability, age‐related morbidities and all‐cause mortality. Its associations with early cognitive changes in midlife is less understood. The aim of this study was to determine whether accelerated PhenoAge in midlife was associated with 10‐year cognitive changes in middle‐aged to older adults.

**Methods:**

This longitudinal study is based on *N* = 2,630 (54% women; mean age 50 years;Table 1) Beaver Dam Offspring Study (BOSS) participants. We measured baseline blood‐based clinical markers of health necessary for calculation of PhenoAge and calculated accelerated PhenoAge (PhenoAgeAccel) as the residual of a linear model with chronological age as the predictor and PhenoAge as the outcome. We tested Trail‐making Test B (TMT‐B) performance at baseline, 5‐year and 10‐year follow‐up. We used a linear mixed‐effects model with PhenoAgeAccel as predictor and TMT‐B time as outcome, adjusting for random intercepts, sex and education. We repeated models stratified by sex.

**Results:**

We found with every additional year older in PhenoAge compared to chronological age at baseline, participants performed worse on the TMT‐B at baseline [complete sample: 0.60 seconds slower, 95% Confidence Interval (0.29,0.91); women: 0.34 (‐0.05,0.72); men: 0.91 (0.41,1.42), Figure 1]. Moreover, with every additional year older in PhenoAge compared to chronological age at baseline, participants had a faster decline in TMT‐B over the 10‐year follow‐up [main effect + wave interaction: complete sample: 0.94 seconds slower (0.58,1.29); women: 0.51 (0.08,0.95); men: 1.48 (0.90,2.07);Figure 1].

**Conclusion:**

Accelerated PhenoAge in midlife was associated with cognitive decline over 10 years, overall and in men. Longer follow‐up will be needed to investigate sex differences further and to determine whether PhenoAge might be predictive of the onset of cognitive impairment later in life. If confirmed, PhenoAge could become a cost‐effective marker of cognitive decline and dementia and might help identify at‐risk individuals early. This could inform targeted prevention and treatment methods to promote healthy brain aging.